# Refractory infantile IPEX with Treg-restricted FOXP3null expression caused by a novel variant in *FOXP3*

**DOI:** 10.70962/jhi.20250249

**Published:** 2026-03-27

**Authors:** Ahmad Rayes, Akshaya Ramachandran, Michael A. Pulsipher, J. Gregory Dolan, Soohee Cho, Amanda K. Johnson, Jessie L. Alexander, Simon Borna, Rosa Bacchetta

**Affiliations:** 1 Pediatric Immunology and Hematopoietic Cell Transplantation/Cellular Therapy Program, Primary Children’s Hospital, University of Utah, Salt Lake City, UT, USA; 2 Division of Hematology, Oncology, Stem Cell Transplantation and Regenerative Medicine, Department of Pediatrics, Stanford University, Stanford, CA, USA; 3 Huntsman Cancer Institute, Spencer Fox Eccles School of Medicine at the University of Utah, Salt Lake City, UT, USA; 4 Institute for Stem Cell Biology and Regenerative Medicine, Stanford University, Stanford, CA, USA; 5 Center for Definitive and Curative Medicine, Stanford University, Stanford, CA, USA

## Abstract

Immune dysregulation, polyendocrinopathy, enteropathy, X-linked (IPEX) syndrome is a life-threatening monogenic inborn error of immunity caused by pathogenic variants in the *FOXP3* gene. In this report, we describe the clinical and immunologic consequences of a novel *FOXP3* variant in an infant with refractory IPEX syndrome. Rapid whole-genome sequencing revealed a missense variant c.1050C>G (p.Ile350Met) in the forkhead domain of *FOXP3*. *FOXP3* expression was abrogated in the regulatory T cell (Treg) subset but was maintained at low levels in activated effector T cells. The frequency of Treg-specific demethylated region (TSDR)–demethylated T cells was within normal range at birth, but it increased to pathologically high levels at 2 wk of age, prior to manifestation of disease symptoms. These results support the clinical relevance of the TSDR-demethylated cell percentage in evaluation of disease activity in IPEX. This case highlights a severe form of IPEX syndrome refractory to multiple immunosuppressive agents and the importance of early immunological studies to verify the clinical significance of novel genetic findings.

## Teaser

This report outlines the clinical, genetic, and immunologic features of a novel FOXP3 missense variant causing severe, treatment-resistant IPEX in an infant, emphasizing the usefulness of quantifying TSDR-demethylated cells as an early biomarker of IPEX disease activity.

## Introduction

Immune dysregulation, polyendocrinopathy, enteropathy, X-linked (IPEX) syndrome is a rare monogenic inborn error of immunity (1). Pathogenic variants in the forkhead (FKH) box P3 (FOXP3) gene cause dysfunction of regulatory T cells (Tregs), resulting in multi-organ autoimmunity such as enteropathy, type 1 diabetes mellitus, and dermatitis (2). Other forms of the disease have been described with moderate, late-onset and chronic evolution involving other organs such as the kidney, thyroid, blood, and liver (2, 3, 4, 5, 6). Untreated severe IPEX is often fatal in the first 2 years of life. Most patients improve with immunosuppressive therapy (IST) in the short term. However, IST often fails to prevent disease progression, and the majority of patients develop complications, leading to poor quality of life and survival. Allogeneic hematopoietic cell transplantation (HCT) is currently the only curative option for IPEX (7).

Genotype–phenotype correlation has been difficult to establish, though mutations resulting in abrogation of FOXP3 protein expression are infrequent and consistently detrimental (1, 8, 9, 10, 11, 12). Similarly, mutations that affect the FKH domain are usually, but not exclusively, responsible for the most severe forms of the disease. However, there is not a consistent clinical phenotype among different patients with the same mutation, including affected siblings. Functional studies are needed to understand the immunological consequences of mutations, but they are difficult to perform, and there are no consensus recommendations for widespread implementation. Since in silico prediction models offer limited clinical guidance, the identification of new *FOXP3* mutations calls for additional tools capable of more accurately forecasting clinical phenotypes and therapeutic responsiveness, ensuring that each patient receives the most appropriate treatment. Quantification of the Treg-specific demethylated region (TSDR)–demethylated T cells in peripheral blood has been reported as a hallmark of the disease, a direct result of the profound immune dysregulatory effect of the FOXP3 mutation in both the Treg and effector T cell (Teff) compartments (11, 13).

Here, we describe a patient with a newly identified mutation, and severe clinical manifestations refractory to treatments, whose severity was directly associated with an elevated percentage of TSDR-demethylated cells that were detected in advance of the onset of the clinical manifestations.

## Results

### Case presentation

A 4-wk-old, full-term, formula-fed, male infant was admitted to the critical care unit with hypovolemic shock in the setting of fever, feeding intolerance, and failure to thrive (FTT). His weight at birth was appropriate for gestational age. He had been intermittently seen by his pediatrician and the emergency room for ongoing concerns of emesis and slow weight gain. Soon after his admission, he developed intractable secretory diarrhea. The diarrhea was characterized by watery, occasionally mucus containing, large volume stools, varying between 60 and 250 ml/kg daily. Although no hematochezia was noted initially, his diarrhea became intermittently bloody. He did not have a clinically significant rash or evidence of dermatitis. His growth was at the third percentile for height (z-score: −1.66) and less than third percentile for weight (z-score: −4.19). His course was further complicated by normocytic anemia, necessitating multiple packed red blood cell transfusions. He also developed central venous catheter-associated deep venous thrombosis, requiring enoxaparin therapy. Postinfectious enteropathy following viral gastroenteritis was suspected initially, and bowel rest was recommended. However, there was no change in his symptoms despite bowel rest and initiation of total parenteral nutrition (TPN). Stool studies did not reveal an infectious etiology but showed elevated fecal calprotectin levels, suggesting intestinal inflammation, which continued to rise over the next few months (Table 1). Exocrine pancreatic function tests revealed increased fecal neutral and split fat along with very low fecal elastase, suggesting severe exocrine insufficiency. He eventually underwent esophagogastroduodenoscopy and sigmoidoscopy at age 8 wk. Gastrointestinal (GI) biopsies showed severe duodenitis with villous atrophy and extensive gastritis with mixed inflammatory infiltrate, including eosinophils along with loss of normal colonic epithelium. Results were suggestive of autoimmune enteritis and colitis, and an immunology consultation was requested. Family history was negative for inborn errors of immunity. Immune function testing results are shown in Table 1 at three time points: 8, 17, and 20 wk of age. The initial consultation was conducted at 8 wk of age, and workup was notable for significantly elevated IgE, decreased FOXP3 expression in Treg (CD4^+^25^+^127^−^) cells and increased proinflammatory cytokines. Rapid whole-genome sequencing was sent to Rady Children’s Institute for Genomic Medicine and revealed a missense variant c.1050C>G (p.Ile350Met) in the FKH domain of FOXP3 predicted by multiple in silico tools to have a deleterious effect on protein function. Although anti-enterocyte antibody testing was not performed, a type 1 diabetes mellitus autoantibody panel was carried out and resulted negative (Table 1).

**Table 1.** Immune function testing results

Test	Reference range	Age 8 wk	Age 17 wk	Age 20 wk
**Complete blood count**				
White blood cell count (K/mcL)	6.5–13.3	14.2	17.5	30.2
Hemoglobin (g/dl)	9.6–12.4	7.3	9.3	9.9
Platelets (K/mcL)	244–529	206	499	342
Absolute neutrophil count (K/mcL)	0.97-5–45	4.5	9.5	28.1
Absolute lymphocyte count (K/mcL)	2.45–8.89	8.4	6.8	1.2
Absolute monocyte count (K/mcL)	0.28-1.07	0.3	1.2	0.9
Absolute eosinophil count (K/mcL)	0.03–0.61	0.7	0	0
**Lymphocyte immunophenotyping by flow cytometry**				
Absolute CD3^+^ T cell count (cells/mcL)	2,200–9,200	5,869	5,674	​
Absolute CD4^+^ count (cells/mcL)	1,600–6,500	3,422	4,260	​
Absolute CD4^+^/CD45RA^+^ count (cells/mcL)	1,200–5,300	2,232	2,371	​
Absolute CD4^+^/CD45RO^+^ count (cells/mcL)	90–1,400	1,303	1,830	​
Absolute CD8^+^ count (cells/mcL)	300–3,400	2,186	1,354	​
Absolute CD19^+^ B cell count (cells/mcL)	520–2,300	401	423	​
Absolute NK cell count (cells/mcL)	97–2,000	555	469	​
**FOXP3 protein expression**				
Treg CD4^+^CD25^+^CD127^−^ (% of CD4)	4.2–9.9	5.5	​	​
Absolute Treg CD4^+^CD25^+^CD127^−^ (cells/mcL)	43–782	143	​	​
Foxp3 (% of Tregs)	55–81	32	​	​
Absolute FOXP3 (cells/mcL)	43–782	46	​	​
**Immunoglobulins**				
IgG (mg/dl)	100–334	205	501	906
IgA (mg/dl)	7–37	18	​	​
IgM(mg/dl)	26–122	19	​	​
IgE (IU/ml)	<=13	9,309	4,997	31,478
**Serum cytokines**				
IL-1 β (pg/ml)	<=6.7	<6.5	<6.5	<6.5
IL-2 (pg/ml)	<=2.1	<2.1	<2.1	<2.1
IL-2 receptor, soluble (pg/ml)	175.3–858.2	6,111.5	3,787	2,322
IL-4 (pg/ml)	<=2.2	<2.2	<2.2	<2.2
IL-5 (pg/ml)	<=2.1	6.1	2.2	<2.1
IL-6 (pg/ml)	<=2.0	49.6	2.8	8.1
IL-8 (pg/ml)	<=3.0	278.1	<3.0	8.4
IL-10 (pg/ml)	<=2.8	48.7	10.6	10.6
IL-12 (pg/ml)	<=1.9	<1.9	<1.9	<1.9
IL-13 (pg/ml)	<=2.3	2.9	<1.7	2.3
IL-17 (pg/ml)	<=1.4	1.5	44.7	12.8
IL-18 (pg/ml)	<=477	1,685	​	​
Interferon-γ (pg/ml)	<=4.2	5.6	<4.2	<4.2
TNF-α (pg/ml)	<=7.2	5.4	2.2	3.8
CXCL9 (pg/ml)	<=647	9,535	​	​
C-reactive protein (CRP) (mg/dl)	<0.3	19.2	5.5	10.9
Erythrocyte Sedimentation rate (ESR) (mm/h)	0–9	14	20	4
Procalcitonin (ng/ml)	0–0.09	0.31	​	14.6
Fecal calprotectin (mcg/g)	​	457	1,200	1,380
Fecal pancreatic elastase (ug/g)	> 200	10	36	​
**Autoantibodies**				
Glutamate decarboxylase antibody (IU/ml)	0–5.0	<5.0 (negative)	​	​
Islet antigen-2 serum autoantibody (unit/ml)	0–7.4	<5.4 (negative)	​	​
Insulin antibody (unit/ml)	0–0.4	<0.4 (negative)	​	​
Islet cell cytoplasmic antibody, IgG	<1:4	<1:4 (negative)	​	​
Zinc transporter 8 antibody (unit/ml)	0–15.0	<10.0 (negative)	​	​

IST with corticosteroids using methylprednisolone and the mTOR inhibitor sirolimus was initiated at 9 wk of age. Sirolimus was selected as first-line treatment given its ability to specifically suppress Teffs while sparing and promoting expansion of Tregs and its reported benefit as a single agent (7, 14). However, persistent vomiting and severe diarrhea prevented achievement of therapeutic drug levels after 2 wk of treatment, likely due to impaired absorption, as sirolimus is only available in oral formulation. Consequently, we transitioned immunosuppression to an intravenously (IV) administered calcineurin inhibitor, tacrolimus. In IPEX syndrome patients, tacrolimus is an effective option for managing autoimmune enteropathy (AIE) by inhibiting T cell activation. Following 3 wk of treatment with IV methylprednisolone and tacrolimus, his symptoms were unchanged with no significant improvement despite achieving therapeutic tacrolimus levels. He continued with intractable large volume secretory diarrhea, intermittent hematochezia, and FTT. Attempts to transition to enteral feeds were unsuccessful, and the patient remained dependent on TPN. Follow up GI biopsies at 12 wk of age showed no histological improvement with findings of ulceration and granulation tissue with no residual epithelium in the stomach or the colon. Tumor necrosis factor-α (TNF-α) inhibition with infliximab was initiated at 14 wk of age due to lack of clinical and histological improvement. Infliximab was selected as a third-line agent given its documented efficacy in inducing remission in refractory AIE. Its use is supported by the targeted inhibition of TNF-α, a key proinflammatory cytokine implicated in the pathogenesis of AIE. Despite a relative reduction in stool volume and hematochezia, along with modest weight gain following several weeks of treatment with infliximab and TPN, his overall clinical condition progressively deteriorated. He continued to experience refractory diarrhea, persistent emesis, and intolerance to enteral feeding. At 16 wk of age, he developed progressive narrowing of the pyloric region, culminating in severe stenosis and gastric outlet obstruction, as demonstrated on upper GI series. Furthermore, progression of his uncontrolled GI inflammation secondary to IPEX-associated AIE was supported by a marked increase in interleukin (IL)-17 levels and fecal calprotectin at 17 wk of age. Due to the patient’s lack of response to prior therapies, abatacept was initiated at 18 wk of age as a fourth-line agent. As a selective negative costimulation modulator, abatacept inhibits T cell activation by mimicking cytotoxic T lymphocyte-associated antigen-4 (CTLA4) competitive binding with CD28 to CD80/86. This approach was guided by emerging evidence supporting its potential efficacy in refractory AIE, including cases associated with IPEX syndrome and other immune dysregulation disorders. However, he remained refractory to treatment, continued to exhibit signs of uncontrolled inflammation, and his symptoms persisted without improvement. HCT was deferred as the patient was considered too ill to safely undergo conditioning, given his poor functional status and multi-organ dysfunction.

Throughout his admission, the patient was found to have cytomegalovirus (CMV) viremia and was treated with ganciclovir and foscarnet. Although CMV colitis was initially suspected, GI biopsies did not demonstrate histopathologic evidence of disease. His CMV viral load improved with therapy, becoming intermittently undetectable and remaining at low levels thereafter. His course was further complicated by multiple culture-negative sepsis episodes and bacterial and viral infections, notably *Staphylococcus aureus* bacteremia and rhinovirus respiratory infection. At 29 wk of age, he developed acute respiratory distress, in the context of progressive abdominal distention, large ascites, and suspected colitis and pancreatitis, as indicated by markedly elevated serum lipase. He was transferred to the pediatric intensive care unit due to clinical deterioration and was presumed to have culture-negative sepsis. Computed tomography demonstrated new diffuse pulmonary opacities bilaterally and a large volume of ascites, with bowel wall thickening and mucosal enhancement. Therapeutic paracentesis was performed to alleviate respiratory distress partially attributable to elevated intra-abdominal pressure from ascitic fluid; however, he later decompensated, necessitating endotracheal intubation and mechanical ventilation. Persistent hypoxemia despite conventional and volume diffusive respirator ventilation led to initiation of veno-venous extracorporeal membrane oxygenation at 30 wk of age, at which time echocardiography showed decreased left ventricular function. Extensive infectious workup was negative, and his progressive respiratory failure and acute respiratory distress syndrome were attributed to an inflammatory pulmonary process. The patient died at 32 wk of age.

### Results of investigational immunological studies

**Figure 1.**
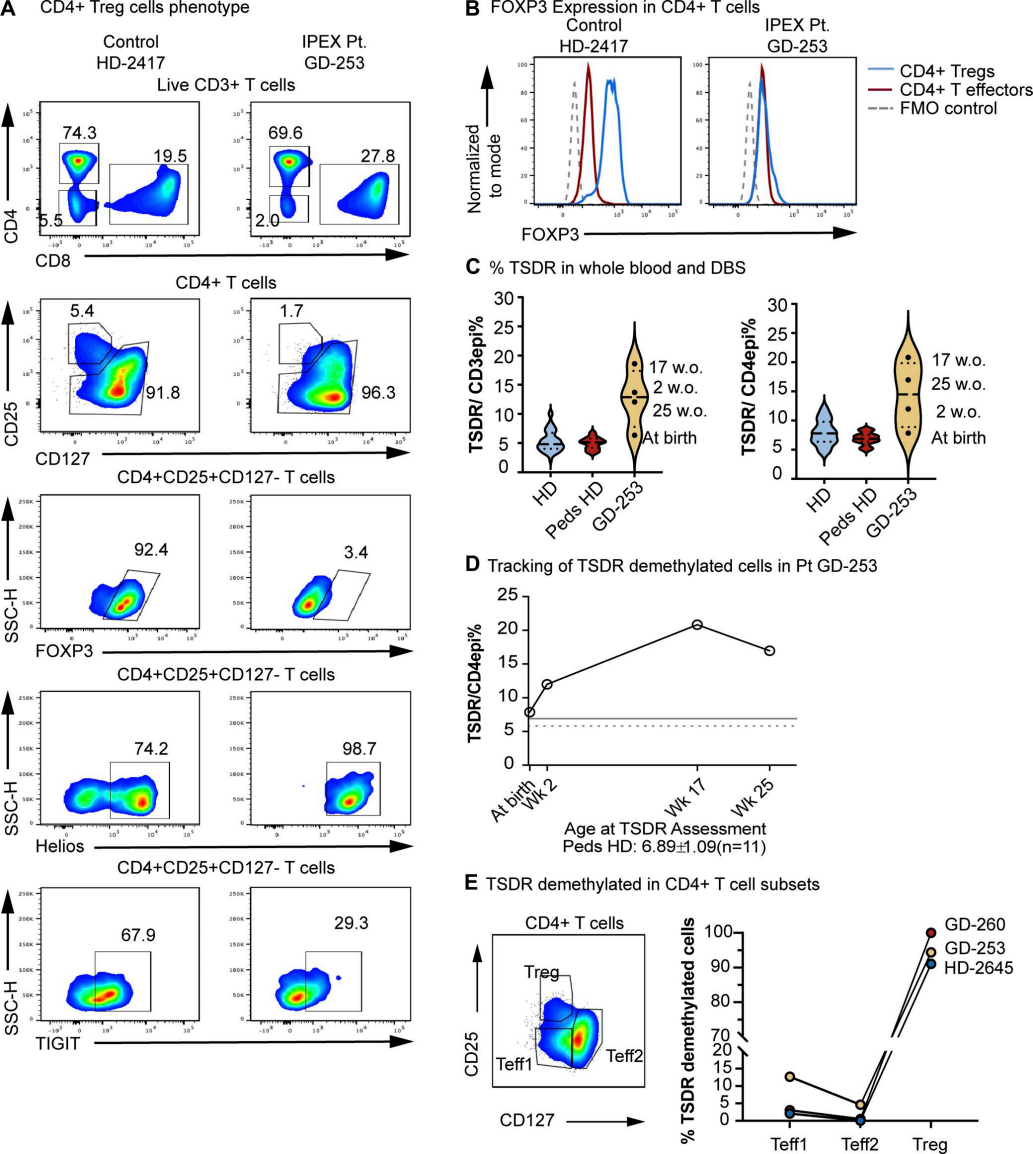
**Patient CD4** ^**+**^ **Tregs demonstrate no meaningful expression of FOXP3. (A)** Flow cytometry analysis of patient PBMC and healthy control show CD4^+^ and CD8^+^ T cells within the CD3^+^ compartment (first row), Treg phenotype (CD25^+^CD127^−^) (second row), expression of FOXP3 (third row), Helios (fourth row), and TIGIT (fifth row) within the Tregs. **(B)** FOXP3 expression in the CD4^+^ Teffs and Tregs, represented as histograms. **(C)** Quantification of TSDR-demethylated cells in CD3^+^ T cells (left) and CD4^+^ T cells (right) from patient samples and male healthy controls (adults, *n* = 33 and pediatric controls, *n* = 11) as well as the patient’s newborn screening dried blood spots (DBSs). **(D)** Kinetics of the percentage of TSDR/CD4 demethylated cells in patient blood since birth. **(E)** Detection of TSDR-demethylated cells within different CD4^+^ T effector compartments. GD-253 (IPEX patient [Pt]), GD-260 (patient’s mother), and HD-2417 and HD-2645 (healthy donors/controls). Gating strategies for Fig 1, A, B, and E are in Fig. S1.

Immunological evaluations performed by the clinical laboratory at week 8 and week 17 (Table 1) revealed a normal absolute number of the main T, B, and natural killer (NK) cell subsets. The percentage and absolute number of CD4^+^CD25^+^CD127^−^ Tregs were low but still within the normal range and with reduced FOXP3 expression. Other immunologic studies at 17 wk showed a very low percentage (1.7%) of CD4^+^CD25^+^CD127^−^ Tregs, which expressed Helios and T cell immunoreceptor with Ig and ITIM domains (TIGIT) but had negligible FOXP3 expression (Fig. 1, A and B).

**Figure S1.**
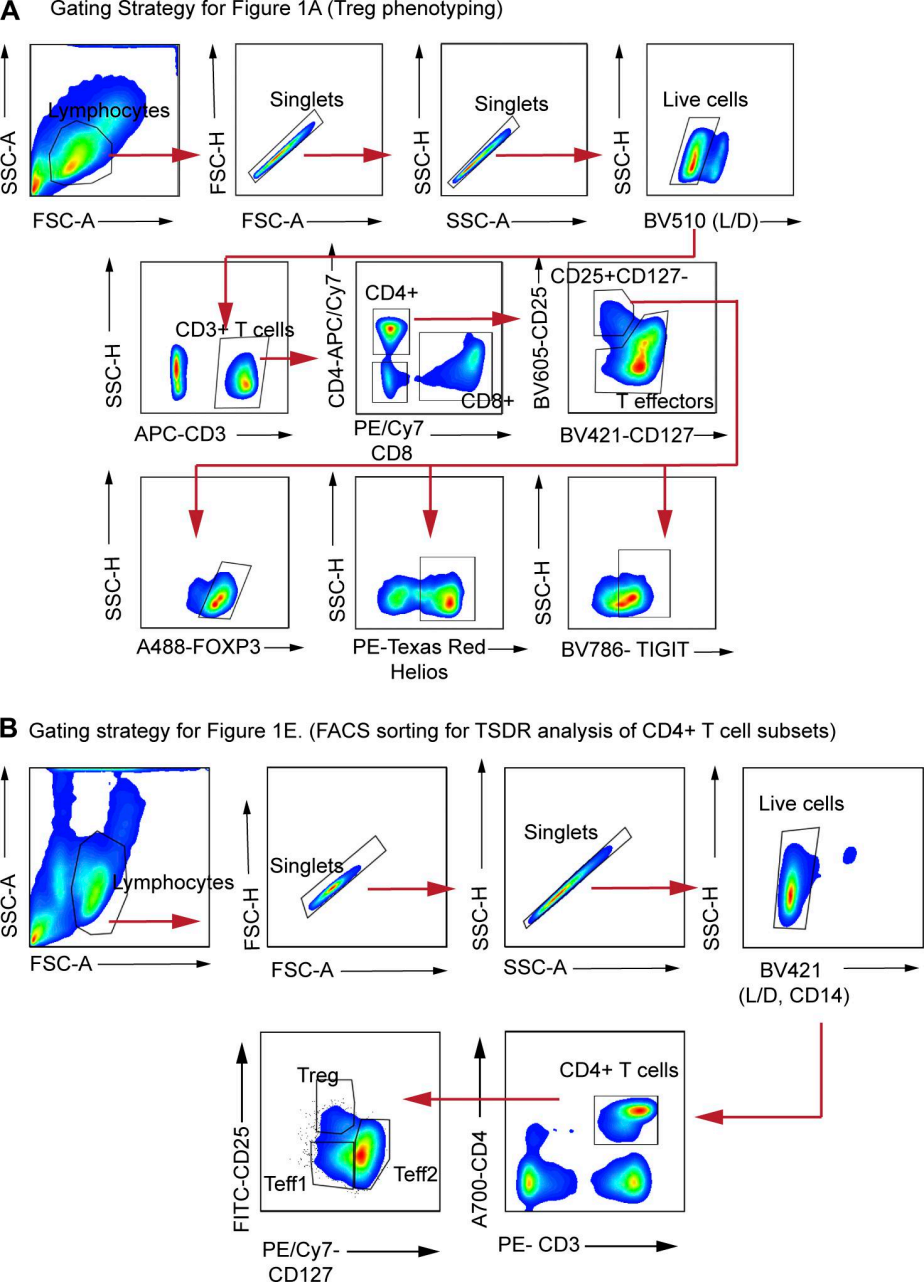
**Gating strategies for Tregs and CD4⁺ T cell subsets.** **(A)** Gating strategy for Treg identification and phenotyping: PBMCs were sequentially gated to exclude debris and doublets, followed by selection of live cells, CD3⁺ T cells, and CD4⁺ T cells. Regulatory T cells (Tregs) were identified as CD25⁺CD127⁻ cells and further characterized by expression of FOXP3, Helios, and TIGIT (Fig. 1, A and B). **(B)** Gating strategy for CD4⁺ T cell subset identification and sorting: After exclusion of debris, doublets, and dead cells, CD3⁺CD4⁺ T cells were gated and subdivided into Treg, Teff1, and Teff2 populations based on CD25 and CD127 expression (Fig. 1 E).

**Figure 2.**
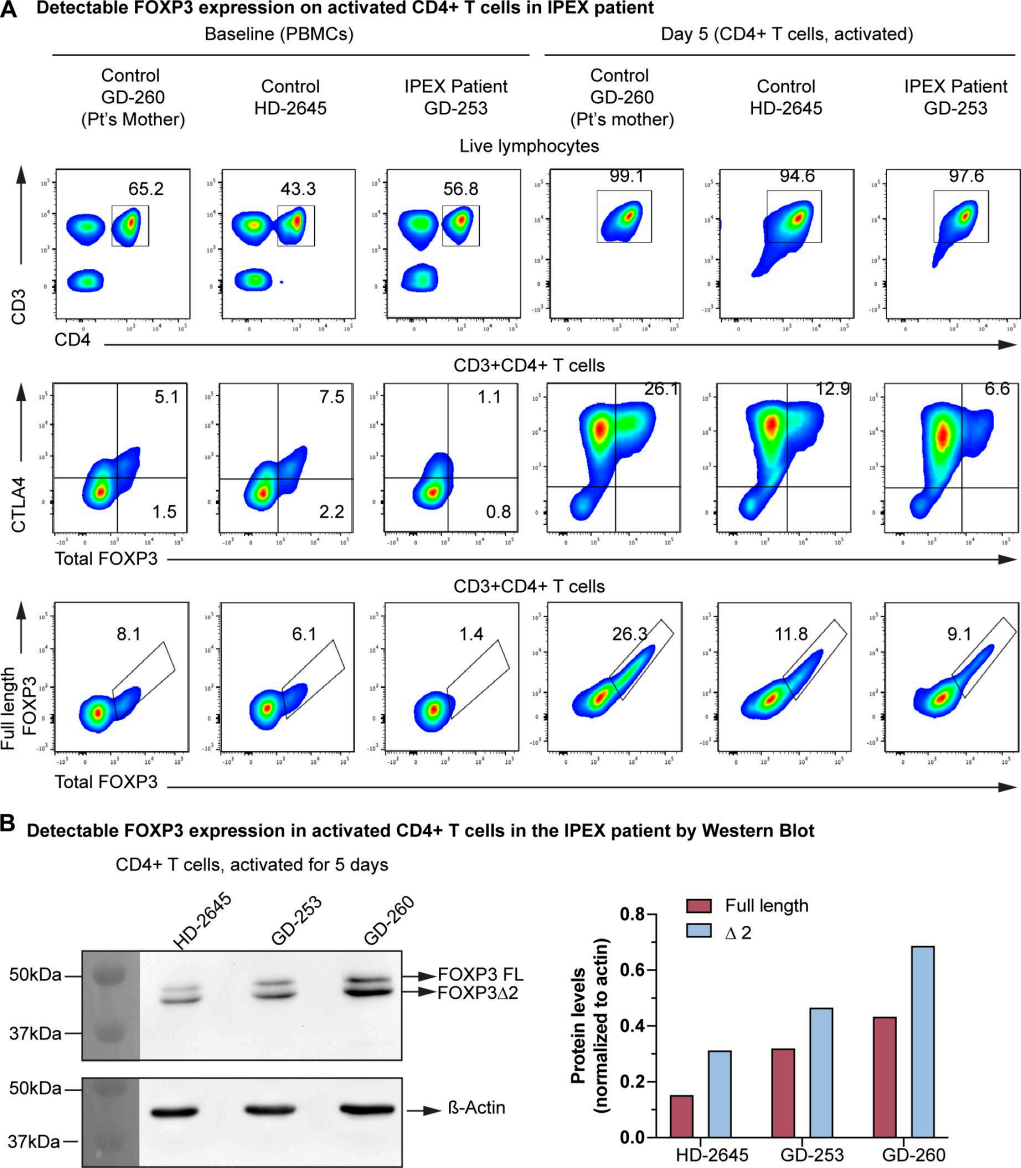
**Activated patient CD4** ^**+**^ **T cells express FOXP3. (A)** Expression of FOXP3 in baseline and activated CD4^+^ T cells (first row) showing total FOXP3 (clone: 259D) and CTLA4 (second row) as well as total FOXP3 (clone: 259D) and full-length FOXP3 (clone: 150D) (third row) expression by flow cytometry in patient’s mother, healthy control, and patient (Pt) CD4^+^ T cells. **(B)** Western blot analysis of FOXP3 expression in the activated CD4^+^ T cells, showing the full-length and delta2 isoforms of the FOXP3 protein along with the quantification of the protein levels. Source data are available for this figure: SourceData F2.

The frequency of TSDR-demethylated T cells quantified from the peripheral whole blood at week 17 and week 25 was consistently elevated as compared to that of healthy adult and pediatric subjects (Fig. 1 C). Retrospective testing of the TSDR frequency in the stored newborn screening cards obtained at birth and at 2 wk of age resulted within the normal range at birth but was already elevated at 2 wk of age, even before the disease manifested clinically (Fig. 1 C). Therefore, the frequency of the TSDR-demethylated cells increased rapidly post-birth (Fig. 1 D). As described in other patients with IPEX, the TSDR-demethylated cells were clearly detected in T effector 1 cells (Teff1) (CD25^−^CD127^−^ cells) and in the T effector 2 cell subsets (CD25^−^CD127^+^), a finding not observed in healthy controls (Fig. S1) (13). Interestingly, FOXP3 expression was abrogated in the (CD4^+^CD25^+^CD127^−^) Treg subset but was maintained at low levels in Teffs after activation, together with expression of other activation markers such as CTLA4 (Fig. 2 A). We also found a normal level of FOXP3 expression within the CD4^+^ T cells of the carrier mother, consistent with the competitive advantage of FOXP3 wild-type Treg over FOXP3-mutated Treg (Fig. 2 A) (15). Induced FOXP3 expression in the activated Teffs allowed us to assess whether the mutation affected expression of the two main FOXP3 isoforms, full-length and delta2, which resulted in both being present at a comparable ratio to that of healthy donors (Fig. 2 B).

**Figure 3.**
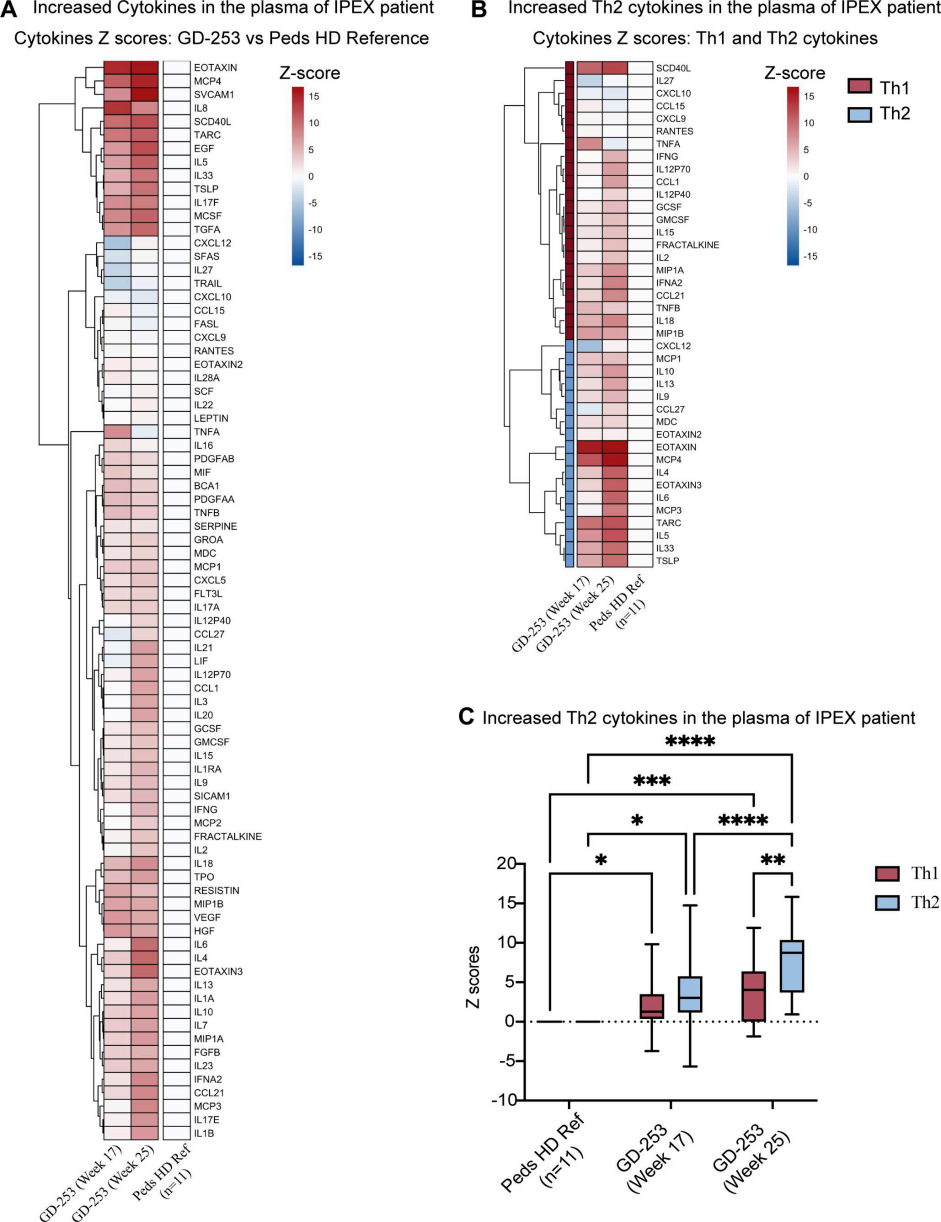
**Elevated cytokine and chemokine profile in the plasma of the patient at week 17 and week 25. (A)** Heatmap representing the plasma cytokine/chemokine levels normalized as Z-scores, compared to healthy control median (male pediatric heathy donors [HD], *n* = 11). **(B)** Heatmap showing the Th1 and Th2 cytokines in the patient plasma. **(C)** Quantification of Th1 and Th2 plasma cytokine and chemokine levels, showing a significant increase in Th2 levels at week 25 compared to week 17 (two-way ANOVA, Šídák's multiple comparisons test; *P < 0.05, **P < 0.01, ***P < 0.001, and ****P < 0.0001).

In accordance with the increased severity of the disease, the plasma cytokines were found to be elevated and increased from week 17 to week 25, revealing predominance of proinflammatory cytokines such as type I and II interferons, IL-6, IL-1α and IL-1β, IL-17, and IL-33 but also a trend to increased T helper 2 (Th2)-type responses as another hallmark of the disease (Fig. 3) (11).

## Discussion

We report the first case of IPEX syndrome in an infant resulting from a novel *FOXP3* missense variant c.1050C>G (p.Ile350Met) in the FKH domain. The clinical presentation and progression in the patient described here are notably among the most severe of reported IPEX cases, both in terms of earlier onset and resistance to therapy. Early reports described successful use of sirolimus in IPEX patients with improvement in enteropathy and reduced corticosteroid dependence, supporting its role as an effective and safe therapeutic option in refractory disease (16). Sirolimus, preferentially inhibits effector T cell proliferation while sparing or promoting Treg survival and function, thereby helping to rebalance immune homeostasis (14, 17). In parallel, translational evidence demonstrates that mTOR inhibition can partially restore Treg-suppressive function in mice and patients with different *FOXP3* mutations through mechanisms independent of FOXP3 expression, further supporting the rationale for mTOR-targeted therapy in IPEX syndrome (18, 19). Our patient, however, did not achieve therapeutic sirolimus levels. Persistent emesis and severe diarrhea likely impaired oral absorption, and as a result, no clinical benefit was observed during the brief treatment period. This case highlights a practical challenge in the use of oral mTOR inhibitors in patients with severe GI involvement. Emerging clinical experience also suggests that abatacept, a recombinant fusion protein of the cell surface marker CTLA-4 and a fragment of immunoglobulin G (CTLA4-Ig) that blocks CD28-mediated costimulation, may hold therapeutic potential in AIE in the setting of IPEX or other immune dysregulation disorders characterized by aberrant T cell activation or lack of regulation (7). It has been shown to prolong survival of FOXP3-deficient mice (20). Case reports have documented meaningful clinical improvement in refractory diarrhea and systemic immune manifestations following abatacept therapy, supporting its use as a targeted immunomodulator when conventional treatments fail (21, 22). One report describes the use of abatacept as part of combination immunomodulatory therapy in a child with severe AIE in the setting of immune dysregulation following neonatal thymectomy (23). The patient’s disease remained refractory despite prompt initiation of multiple immunosuppressive agents and continued to exhibit uncontrolled inflammation leading to his death at 32 wk of age, which reflects the severity of his clinical phenotype.

The c.1050C>G (p.Ile350Met) variant has not been previously described in humans. However, isoleucine 350 is conserved between mouse and human (24), and its substitution with asparagine has been shown to cause a scurfy-like phenotype in mice (25). In addition, Ho-Keun Kwon et al. transduced CD4^+^ T cells with a Foxp3 cDNA in which isoleucine 350 was substituted with alanine and showed that this substitution leads to loss of Foxp3 expression (26). Notably, isoleucine 350 was mutated to two different amino acids with distinct biochemical properties, yet these substitutions had a clear impact on the mouse phenotype and Foxp3 expression in CD4^+^ T cells. Consistent with our data, these findings strongly suggest that substitutions of isoleucine 350 are likely to impact Foxp3 expression and function. Although genotype–phenotype correlation in IPEX is challenging to establish, all patients with complete loss of FOXP3 protein expression have presented with the classical, severe, early-onset form of the disease (8, 9, 10). Uniquely, in this patient’s CD4^+^ T cells, FOXP3 expression was specifically abrogated in the Tregs but detectable upon activation in Teffs. This difference confirms the differential regulation of FOXP3 expression in Tregs and Teffs. In addition, the CD25^high^ CD127^low^ phenotypic Treg compartment of the FOXP3 mutation carrier mother contains almost exclusively FOXP3^+^ cells, demonstrating the competitive advantage of the Tregs expressing the wild-type FOXP3. The carrier mother showed normal distribution of TSDR-demethylated cells, thus excluding the possibility that the Tregs expressing the mutated FOXP3 are present outside of the phenotypic Treg compartment. These data are consistent with our previous report showing the competitive advantage of FOXP3 mutant over wild-type Tregs (15).

IPEX, although rare, should be considered promptly in a male patient with early-onset severe enteropathy and FTT. Over 187 different *FOXP3* pathogenic variants have now been described in patients with IPEX syndrome, most residing in sequence encoding the C-terminal FKH DNA-binding domain (1, 2, 12). However, about 300 different mutations are scattered across the *FOXP3* gene in patients with the IPEX phenotype. Interpreting a newly identified mutation can be challenging with respect to diagnosis, prognosis, and treatment options.

In this case, we emphasize the importance of quantifying the frequency of TSDR-demethylated cells, as elevated levels may indicate a severe IPEX phenotype (11, 13). Demethylation of Treg-specific CpG islands within the conserved noncoding sequence 2 of the *FOXP3* gene—known as the TSDR—serves as a lineage-specific epigenetic marker essential for stable *FOXP3* expression (11). TSDR demethylation represents an epigenetic signature of thymic Tregs, distinguishing bona fide Tregs, which are fully demethylated at this site, from activated Teffs and other blood cell types that retain methylation of the *FOXP3* TSDR (11, 13). The proportion of cells exhibiting TSDR demethylation is considered an independent biomarker of IPEX. Specifically, the ratio of *FOXP3* TSDR-demethylated cells to CD4-demethylated cells (%TSDR/CD4) has been shown to effectively discriminate patients with IPEX from unaffected controls (27). In this patient presented here, the markedly elevated proportion of demethylated CD4^+^ T cells predicted a severe phenotype even before the onset of clinical symptoms. TSDR demethylation enables enumeration of Treg-lineage cells independently of phenotype (28, 29, 30, 31). In IPEX, elevated TSDR-demethylated cell frequencies occur despite normal to low phenotypic CD25highCD127low Tregs (11, 13, 32, 33). This discrepancy reflects “loss-of-identity” Tregs—FOXP3-mutated cells that lose phenotypic markers and FOXP3 expression while preserving TSDR demethylation and acquiring effector-like features (13). These unstable Tregs expand in response to autoantigen stimulation and contribute to disease progression, with expansion more pronounced when FOXP3 function is severely impaired (11, 13). In this patient, the rapid rise in TSDR demethylation from normal at birth to elevated at 2 wk—preceding clinical symptoms—exemplifies how TSDR elevation reflects expansion and loss of identity of Tregs during active autoimmune disease.

Our findings reinforce the clinical utility of quantifying TSDR-demethylated cells as a complementary tool to genetic analysis, aiding accurate diagnosis, and providing insights into disease severity and activity. We have previously shown that a high frequency of TSDR-demethylated cells reflects an increased number of cells that retain the Treg lineage marker but lose their typical phenotype and function. These cells, commonly found within the Teff1 subset as in this patient, acquire effector-like properties, including enhanced cytokine production—particularly GM-CSF and Th2-type cytokines—detected in this patient’s plasma, thereby supporting the diagnosis (11, 13). Evaluating TSDR relative to CD4^+^ T cell frequency is critical, as the Treg-to-Teff ratio is essential for immune homeostasis, and absolute TSDR values can be influenced by lymphopenia. The TSDR/CD4 ratio provides a more accurate assessment by excluding falsely low or elevated values due to changes in total T cell counts. When TSDR values overlap with healthy donors, interpretation may be challenging; for novel variant of uncertain significance without clinical manifestations, repeat testing is warranted as disease onset may occur later or remain mild as seen in atypical IPEX (11). In symptomatic patients with known pathogenic mutations, normal TSDR values may rarely reflect acquired lymphopenia from prolonged immunosuppression. The TSDR assay is well-established and highly sensitive, with ISO15189 accreditation in Europe for IPEX diagnosis. Because quantification is DNA-based, results are minimally affected by sample processing, can be obtained from dried blood spots, and remain stable across pediatric and adult age groups (11, 32).

In summary, our case highlights a novel FHK-domain missense *FOXP3* variant leading to a severe form of IPEX syndrome refractory to several immunosuppressive agents and emphasizes the importance of utilizing early immunological studies, including quantitating *FOXP3* TSDR demethylated cells, to complement gene sequencing in the diagnosis of IPEX.

## Materials and methods

### Sample collection

Peripheral blood from the patient and his mother were collected in EDTA tubes in accordance with BMT Protocol Number 328 of the Center for Genetic Immune Diseases, approved by the Stanford University Institutional Review Board. Dry blood spots from the patient were collected at the time of newborn screening and made available by the Department of Health and Human Services. Healthy control peripheral blood was obtained from the Stanford Blood Bank.

### Peripheral blood mononuclear cell (PBMC) and CD4^+^ T cells isolation and activation

PBMCs were isolated from peripheral whole blood through density gradient separation using GE Healthcare Ficoll-Paque PLUS media (17144003). The freshly isolated PBMCs were used for flow cytometry analysis, CD4^+^ T cell isolation, and activation. Remaining cells were frozen in CryoStor CS10 media (1001061; STEMCELL Technologies) for future use.

CD4^+^ T cells were isolated from freshly isolated PBMC using human CD4^+^ T cells Isolation kit (130-096-533; Miltenyi) according to the manufacturer’s instructions. The isolated CD4^+^ T cells were activated with Human T-Activator anti-CD3/CD28 dynabeads (11131D; Gibco) at 1:10 bead-to-cell (b:c) ratio for 5 days in X-VIVO 15 media (02-060Q; Lonza) supplemented with 5% Human Serum (H3667; Sigma-Aldrich).

### Flow cytometry

Treg immunophenotyping was performed on PBMCs in staining buffer with the following surface antibodies: CD3 (Cat# 317318; BioLegend, RRID:AB_1937212), CD4 (Cat# 300518; BioLegend, RRID:AB_314086), CD25 (at# 562660; BD Biosciences, RRID:AB_2744343), CD127 (Cat# 351310; BioLegend, RRID:AB_10960140), and TIGIT (Cat# 372735; BioLegend, RRID:AB_2892455), and intracellular antibodies: FOXP3 (Cat# 320212; BioLegend, RRID:AB_430887) and Helios PE/Dazzle (Cat# 137232; BioLegend, RRID:AB_2565797) after fixation and permeabilization using eBioscience Foxp3/Transcription Factor Staining Buffer Set (50-112-8857; Thermo Fisher Scientific). The activated CD4^+^ T cells were stained with the surface antibodies: CD3, CD4, and CD25, and intracellular antibodies: FOXP3 (Cat# 320212; BioLegend, RRID:AB_430887; Cat# 320008; BioLegend, RRID:AB_492980) and CTLA-4 (Cat# 349908; BioLegend, RRID:AB_10679122. For FACS sorting of CD4^+^ T cell subsets, the CD4^+^ T cells were stained with surface antibodies: CD3 (Cat# 317308; BioLegend, RRID:AB_571913), CD4 (Cat# 561030; BD Biosciences, RRID:AB_10563215), CD14 (Cat# 301828; BioLegend, RRID:AB_2275670), CD25 (Cat# 347643; BD Biosciences, RRID:AB_400334), and CD127 (Cat# 351320; BioLegend, RRID:AB_10897098). Data were acquired on BD FACSAria II Cell Sorter (RRID:SCR_018934) and 5-laser BD FACSymphony A5 Cell Analyzer (RRID:SCR_022538), and data analysis was performed using FlowJo software version 10 (RRID:SCR_008520).

### Western blot

Activated CD4^+^ T cells were lysed with a lysis buffer cocktail consisting of SDS Lysis Buffer, 50 mM DTT, and 1X Protease Inhibitor cocktail. The lysates were then loaded and resolved using 11% SDS-PAGE. The membrane was developed with an anti-human FOXP3 primary antibody (Cat# 320202; BioLegend, RRID:AB_430885) and loading control HRP-conjugated β-actin antibody (Cat# sc-47778 HRP; Santa Cruz Biotechnology, RRID:AB_2714189). Membranes were imaged with Bio-Rad ChemiDoc Imager, and data analysis was performed using ImageJ software (Rasband, W. S., ImageJ, U. S. National Institutes of Health, Bethesda, MD, USA, https://imagej.nih.gov/ij/, 1997–2018, RRID:SCR_003070).

### TSDR analysis using epigenetic quantitative PCR testing protocol

Peripheral whole blood or dried blood spots were lysed at 56°C for 15 min. DNA isolation and bisulfite conversion were performed using BisuPrep Reagents (RUO) from Epimune Diagnostics. The bisulfite converted DNA was similarly isolated from sorted FACS sorted cell pellets. The eluted DNA was then used to perform quantitative PCR using specific markers for FOXP3+ Tregs, CD3^+^ T cells, CD4^+^ T cells, and glyceraldehyde-3-phosphate dehydrogenase (GAPDH), and the quantification of cell subsets was performed as already described (11, 13, 34).

### Plasma luminex assay

Luminex H80 multiplex assay was performed on frozen plasma samples obtained from EDTA blood tubes using Luminex xMAP technology at the Stanford Human Immune Monitoring Center. Artifacts caused by batch, plate, and nonspecific binding were removed as previously described (11). The Luminex mean fluorescence intensity (MFI) values for the cytokines and chemokines of the patient sample were compared to the median values from the lab reference dataset of healthy male pediatric donors (HD) (ages 2–17) (*n* = 11) to calculate a z-score using the formula:

$$z-score=\frac{Patient\;MFI-HD\;MFI\;median}{HD\;MFI\;median\;absolute\;deviation}$$

Statistical analyses were conducted using R (Version 4.4.3). The code for the cytokine analysis and heatmap generation is archived on Zenodo (https://doi.org/10.5281/zenodo.17666405).

## Data availability 

The data underlying Figs. 1, 2, and 3 are available in the published article and its online supplemental material.

## Supplementary Material

SourceData F2 is the source file for Fig. 2.
