# Supplementary material for: Refractory infantile IPEX with Treg-restricted FOXP3null expression caused by a novel variant in FOXP3
Source: J Hum Immun. 2026 Mar 27;2(3):e20250249. doi: 10.70962/jhi.20250249 (PMC13023379; doi:10.70962/jhi.20250249)

B. Detectable FOXP3 expression in activated CD4+ T cells in the IPEX patient by Western Blot

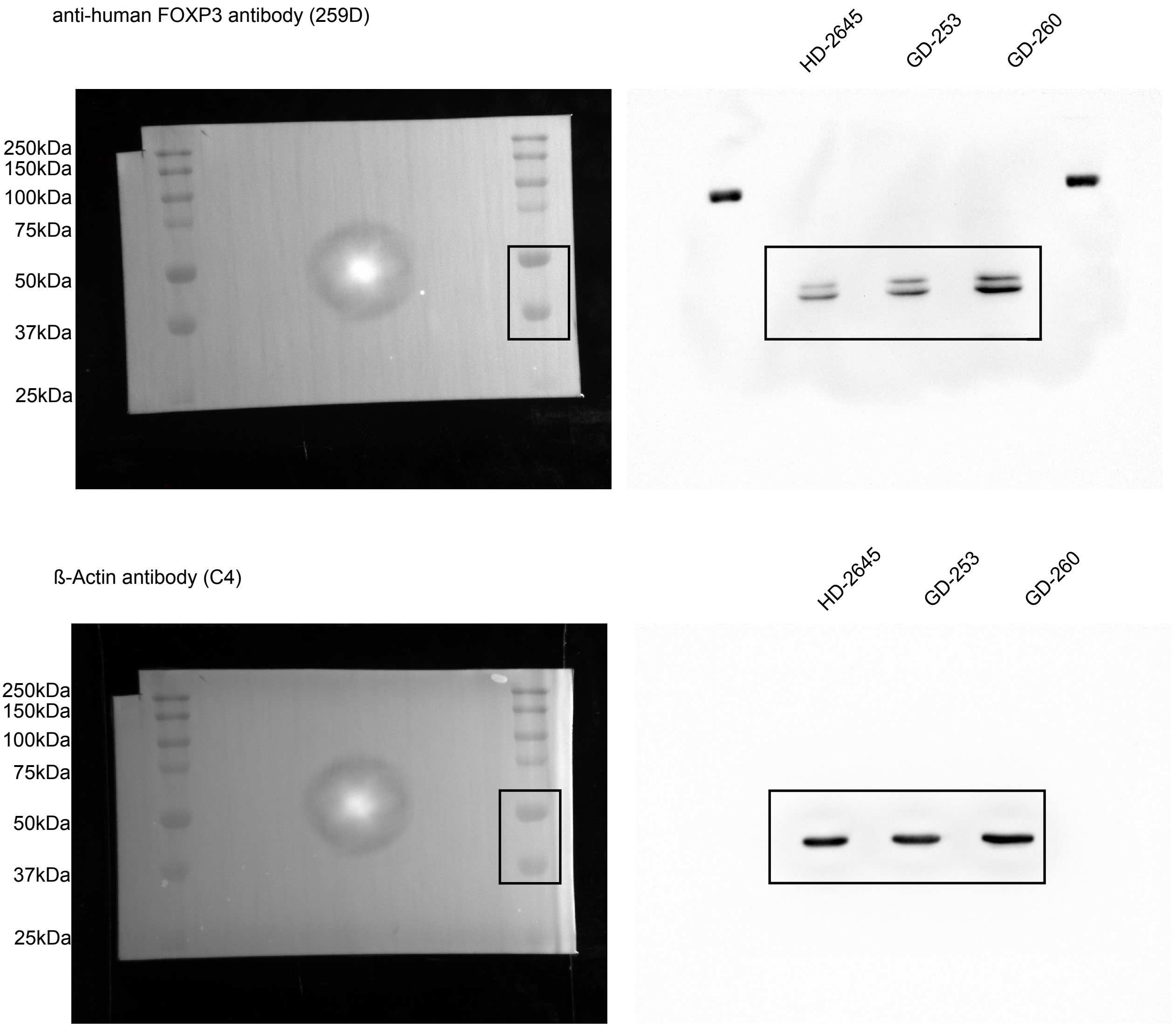

Supplement: SourceData F2 — is the source file for Fig. 2. [file jhi_20250249_sourcedataf2.pdf]
